# Dual Compartmentalization of GSTA4 Suppresses Ferroptosis to Drive Antiandrogen Resistance in Prostate Cancer

**DOI:** 10.1002/advs.77908

**Published:** 2026-09-27

**Authors:** Yong Luo, Wei Zhuang, Zean Li, Yiming Lai, Yongyi Ou, Dongquan Li, Shanhe Huang, Tianlong Luo, Jintao Hu, Bingliang Chen, Jianhan Fu, Tao Zhuo, Hankun Jiang, Yin Lu, Hai Huang, Kewei Xu, Shengmeng Peng, Qing Yuan

**Affiliations:** ^1^ Department of Urology Sun Yat‐sen Memorial Hospital Sun Yat‐sen University Guangzhou China; ^2^ Department of Urology The First Affiliated Hospital Hengyang Medical School University of South China Hengyang China; ^3^ Department of Urology The Second Affiliated Hospital of Fujian Medical University Quanzhou China; ^4^ Guangdong Provincial Key Laboratory of Malignant Tumor Epigenetics and Gene Regulation Sun Yat‐Sen Memorial Hospital Sun Yat‐Sen University Guangzhou China; ^5^ Guangdong Provincial Clinical Research Center for Urological Diseases, Sun Yat‐Sen Memorial Hospital Sun Yat‐Sen University Guangzhou China; ^6^ Vancouver Prostate Centre Department of Urologic Sciences University of British Columbia Canada; ^7^ The Fifth Affiliated Hospital of Xinjiang Medical University Urumqi China; ^8^ Senior Department of Urology Chinese PLA General Hospital Beijing China; ^9^ Department of Urology The Sixth Affiliated Hospital of Guangzhou Medical University Qingyuan People's Hospital Qingyuan China; ^10^ Southern Medical University Guangzhou China; ^11^ Senior Department of Urology The Third Medical Centre of Chinese People's Liberation Army (PLA) General Hospital Beijing China

**Keywords:** enzalutamide resistance, GSTA4, mitochondrial fission, non‐canonical ferroptosis, prostate cancer

## Abstract

Resistance to second‐generation antiandrogens poses a major therapeutic challenge in castration‐resistant prostate cancer (CRPC). While ferroptosis evasion has been implicated in treatment failure, the key molecular determinants driving this evasive process are poorly understood. Using integrated multi‐omics analyses of resistant models and patient specimens, we identify glutathione S‐transferase alpha 4 (GSTA4) as a pivotal ferroptosis suppressor that is associated with adverse clinical outcomes and drives antiandrogen resistance via a dual compartmentalization mechanism. Our data demonstrate that GSTA4 not only detoxifies the lipid peroxidation product 4‐hydroxynonenal in the cytosol but also translocates to the mitochondria under ferroptotic stress. GSTA4 interacts with PGAM5 to prevent the dephosphorylation of Drp1 at Ser637, thereby maintaining mitochondrial fitness and suppressing ferroptosis. This spatially coordinated defense program is crucial for antiandrogen resistance, as GSTA4 knockdown sensitizes tumors to antiandrogen therapy. Virtual screening and surface plasmon resonance assays identify metformin as a candidate GSTA4 inhibitor. Targeting GSTA4 with metformin restores ferroptosis sensitivity and reverses antiandrogen resistance both in vitro and in vivo. Our findings uncover a spatially coordinated anti‐ferroptotic mechanism underlying antiandrogen resistance and highlight targeting GSTA4 as a promising combination strategy for CRPC treatment.

## Graphical abstract

The schematic diagram depicts a compartmentalized anti‐ferroptotic defense mechanism mediated by GSTA4. GSTA4 is upregulated and localizes to the cytoplasm in ENZR cells, where it detoxifies 4‐HNE via a non‐canonical GSH‐dependent pathway. Under oxidative stress, GSTA4 translocates to mitochondria, interacts with PGAM5, and blocks Drp1 Ser637 dephosphorylation, thereby preserving mitochondrial fitness. This coordinated cytoprotective response confers antiandrogen resistance, which can be therapeutically reversed by the GSTA4 inhibitor metformin to induce ferroptosis.

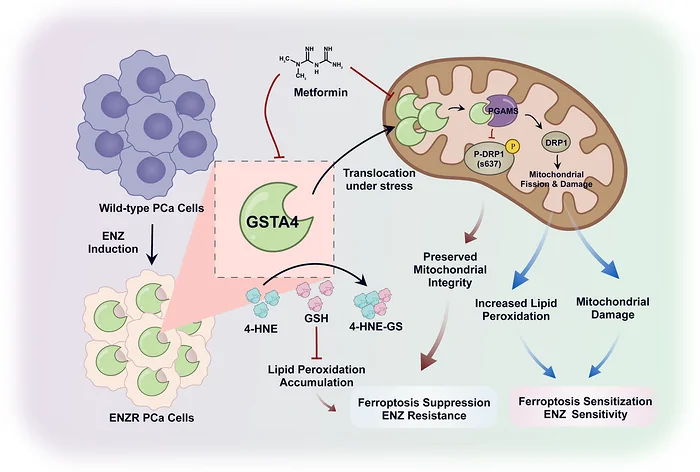

## 1 Introduction

Prostate cancer (PCa) progression to castration‐resistant prostate cancer (CRPC) is frequently driven by sustained androgen receptor (AR) signaling and profound metabolic reprogramming [1, 2, 3]. Second‐generation antiandrogen therapies (SGAT), including enzalutamide (ENZ) and abiraterone, serve as standard treatments for advanced PCa [4, 5, 6]. However, resistance to these agents inevitably emerges, highlighting the urgent need to define AR‐independent mechanisms that sustain tumor survival [7, 8].

Recent studies have implicated a connection between ENZ resistance and ferroptosis, a regulated form of cell death triggered by iron‐dependent lipid peroxidation [9, 10]. Ferroptosis is primarily governed by the canonical glutathione (GSH)‐glutathione peroxidase 4 (GPX4) axis, in which GPX4 utilizes GSH to detoxify lipid hydroperoxides and prevent lethal peroxidation [11]. Accumulating evidence underscores a pivotal role for ferroptosis in mediating resistance to multiple anticancer agents [12, 13, 14]. In CRPC, resistance to standard therapies such as ENZ is almost inevitable, and ferroptosis susceptibility appears to be extensively rewired [9, 15]. Recent reports suggest that ENZ treatment remodels lipid metabolism, thereby enhancing sensitivity to GPX4 inhibition and ferroptosis [16, 17].

While the canonical GPX4‐dependent pathway is well‐established as a central regulator of ferroptosis, emerging evidence highlights the physiological relevance of non‐canonical regulatory mechanisms [18, 19]. Although non‐GPX4‐mediated pathways are increasingly recognized as contributors to therapy resistance across various cancers [20, 21], their specific involvement in CRPC remains largely unexplored. Evidence from diverse malignancies indicates that drug resistance often involves metabolic adaptations, notably lipid metabolic rewiring, along with the activation of non‐canonical cytoprotective programs that mitigate ferroptotic stress [22, 23]. However, the emergence of therapeutic resistance implies the activation of adaptive compensatory pathways, yet the underlying mechanisms, particularly those independent of GPX4, remain poorly defined, thereby hampering the development of effective ferroptosis‐targeting strategies to overcome drug resistance in CRPC.

Beyond the canonical ferroptosis regulators, the broader landscape of redox‐homeostatic enzymes that may be co‐opted to promote survival under therapeutic pressure remains inadequately charted. Among these enzymes, glutathione S‐transferase alpha 4 (GSTA4) has been implicated in maintaining cellular redox homeostasis [24, 25]. Accumulating evidence suggests that GSTA4 may contribute to tumor progression and therapy resistance across various cancer types by mitigating oxidative damage [26, 27]. Notably, the role of GSTA4 in ferroptosis regulation has not been systematically investigated, particularly in the context of CRPC progression. Thus, the biological significance and molecular basis of GSTA4 in CRPC ferroptosis regulation warrant further elucidation.

In this study, we establish that ENZ‐resistant CRPC cells exhibit profound dysregulation of lipid peroxidation and ferroptosis evasion. Transcriptomic and functional screens identify GSTA4 as a central ferroptosis suppressor driving ENZ resistance in CRPC. GSTA4 limits cytosolic lipid peroxidation and, after mitochondrial translocation, preserves mitochondrial integrity by preventing PGAM5‐mediated Drp1 dephosphorylation. This dual protective mechanism enables ferroptosis evasion. Metformin binds to GSTA4 and inhibits its enzymatic activity, thereby disrupting this compensatory pathway, restoring ferroptosis sensitivity, and reversing ENZ resistance both in vitro and in vivo.

## 2 Results

### 2.1 Ferroptosis Suppression is a Hallmark of ENZ‐Resistant CRPC

**FIGURE 1:**
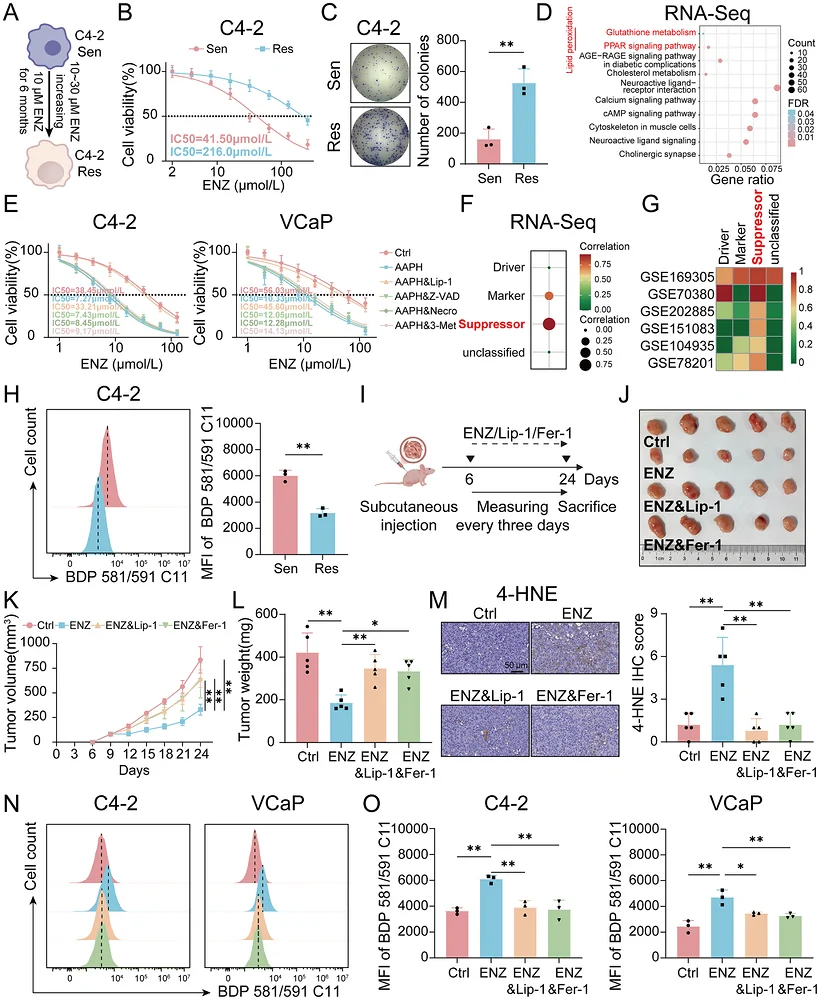
Ferroptosis suppression is a hallmark of ENZ‐resistant CRPC. (A) Schematic workflow for the generation of C4‐2‐ENZR cell line. (B) ENZR cells exhibited a significantly higher IC_50_ for ENZ compared to that of parental cells. (C) Colony formation assay demonstrated enhanced clonogenic survival of ENZR cells upon ENZ treatment. (D) Functional enrichment analysis of our ENZR transcriptomic data revealed significant enrichment of pathways involved in lipid peroxidation. (E) Assessment of cell death patterns under ENZ treatment confirmed that only ferroptosis inhibitors significantly attenuated AAPH‐induced lipid peroxidation. (F) ssGSEA of our ENZR transcriptomic data using FerrDb gene sets demonstrated pronounced enrichment of ferroptosis‐suppressive pathways in resistant tumors. (G) ssGSEA analysis of six ENZ‐resistant datasets from the GEO database using FerrDb gene sets confirmed consistent enrichment of ferroptosis‐suppressive signatures. (H) ENZR cells showed reduced basal lipid peroxidation levels, as measured by BODIPY 581/591 C11 fluorescence. (I) Schematic illustration of the in vivo xenograft experiment assessing the therapeutic efficacy of combining ENZ with ferroptosis inhibitors (Lip‐1 and Fer‐1). (J) Representative photographs of resected xenograft tumors from each treatment group. (K, L) Tumor growth curves (K) and tumor weights (L) show corresponding changes across the different treatment groups. (M) IHC assay for the lipid peroxidation marker 4‐HNE in xenograft tissues. Scale bar, 50 µm. (N, O) Flow cytometry analysis confirmed that Lip‐1 and Fer‐1 inhibited ENZ‐induced lipid peroxidation in vitro.

To delineate the mechanisms underlying ENZ resistance, we established ENZ‐resistant (ENZR) C4‐2 cells (Figure 1A), which displayed increased viability and colony formation upon ENZ treatment (Figure 1B,C). Transcriptomic profiling (Figure S1A) revealed significant enrichment of pathways converging on lipid peroxidation, including glutathione metabolism and PPAR signaling (Figure 1D), consistent with a well‐established mechanism underlying therapy resistance [9]. Furthermore, analysis of six independent ENZR transcriptomic datasets from the GEO database (Figure S1B–G) corroborated this metabolic rewiring (Figure S1H–M), reinforcing the central role of lipid peroxidation. We next treated the CRPC cell lines C4‐2 and VCaP with the lipid peroxidation inducer AAPH under ENZ challenge, which sensitized cells to ENZ (Figure 1E). Importantly, this sensitizing effect was completely abolished by ferroptosis inhibition (Figure 1E), indicating that ferroptosis contributes substantially to ENZ sensitivity. To further evaluate the association between ENZ resistance and ferroptosis, we performed ssGSEA analysis using FerrDb‐defined ferroptosis‐related gene sets [28] along with our ENZR transcriptomic data. The analysis revealed a pronounced enrichment of ferroptosis‐suppressive signatures (Figure 1F), which was validated in the above six ENZ‐resistant datasets (Figure 1G). Subsequent lipid peroxidation assays consistently confirmed reduced lipid peroxidation levels in resistant cells (Figure 1H). Consistent with these findings, basal lipid peroxidation was significantly reduced in ENZR cells, as evidenced by lower 4‐HNE levels, whereas intracellular GSH levels were markedly increased after normalization to cell numbers (Figure S1N,O), further supporting that ENZ‐resistant cells maintain an intrinsically enhanced antioxidant state.

To elucidate whether ferroptosis suppression promotes ENZ resistance, we conducted functional assays using both in vitro and in vivo models. Notably, administration of the ferroptosis inhibitors significantly reversed the ENZ‐induced decrease in tumor volume and growth in xenograft models without affecting body weight (Figure 1I–L and Figure S1P) and reduced ENZ sensitivity in vitro (Figure S1Q,R). Immunohistochemical (IHC) analysis of tumor sections demonstrated that these inhibitors substantially attenuated the ENZ‐induced upregulation of 4‐HNE, a key lipid peroxidation marker (Figure 1M). Subsequent flow cytometry using the BODIPY‐C11 probe further confirmed that ferroptosis inhibition markedly suppressed ENZ‐triggered lipid peroxidation (Figure 1N,O). Collectively, these findings reveal that acquired resistance to ENZ is accompanied by a reduction in lipid peroxidation and an ensuing suppression of ferroptosis.

### 2.2 GSTA4 is a Key Ferroptosis Suppressor Upregulated in ENZR CRPC and is Associated With a Poor Prognosis

**FIGURE 2:**
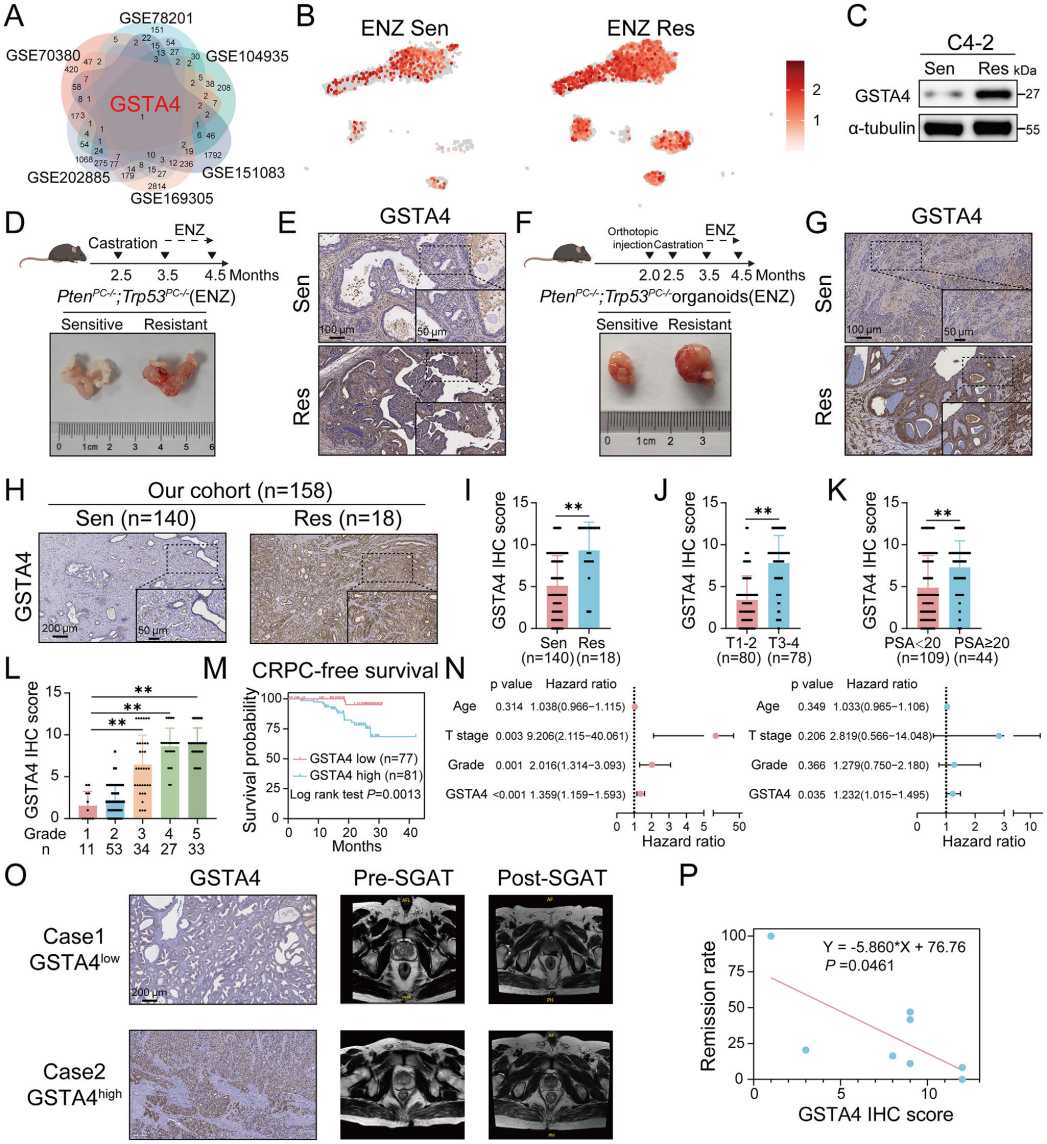
GSTA4 is a key ferroptosis suppressor upregulated in ENZR CRPC and predicts poor prognosis. (A) Intersection analysis of DEGs from six ENZ‐resistant GEO datasets (visualized by Venn diagram) identified GSTA4 as a consensus gene associated with ENZ resistance. (B) ScRNA‐seq analysis (GSE206962) revealed enriched expression of GSTA4 specifically in tumor cells of ENZR patients (right) compared to those from ENZ‐sensitive counterparts (left). (C) Western blot analysis confirmed GSTA4 upregulation in our ENZR cells. (D) Schematic illustration of the ENZR CRPC mouse model established using *Pten/Trp53* double‐knockout mice. (E) IHC confirmed GSTA4 upregulation in ENZR tumors from the spontaneous CRPC model. Scale bars: 100 µm and 50 µm (insets). (F) Schematic illustration of the orthotopic ENZR CRPC model established with *Pten/Trp53*‐deficient prostate organoids. (G) IHC confirmed GSTA4 upregulation in ENZR tumors from the orthotopic organoid model. Scale bars: 100 µm and 50 µm (insets). (H, I) Representative GSTA4 IHC images (H) and quantification (I) in a clinical cohort (n = 158) showed elevated expression in tumors resistant to SGAT. Scale bars: 200 and 50 µm (insets). (J‐L) GSTA4 expression was significantly associated with advanced tumor stage (J), elevated PSA levels (K), and a higher tumor grade (L) in our cohort. (M) Kaplan‐Meier analysis showed significantly reduced CRPC‐free survival in patients with high GSTA4 expression. (N) Univariate and multivariate Cox regression analysis for CRPC‐free survival identified GSTA4 as an independent prognostic factor. (O, P) Correlation analysis of paired representative pre‐ and post‐SGAT MRI images with GSTA4 IHC scores revealed an inverse correlation between GSTA4 expression and therapeutic efficacy. Scale bar: 200 µm.

To delineate core ferroptosis suppressors driving CRPC progression, we integrated gene expression profiles from ENZR cell lines across multiple GEO datasets. Intersection analysis of differentially expressed genes (DEGs) identified GSTA4 as a consistently upregulated candidate (Figure 2A). Notably, GSTA4 was consistently upregulated in ENZR datasets, as underscored by radial plot analysis (Figure S2A). scRNA‐seq of human ENZ‐resistant and ENZ‐sensitive PCa samples (GSE206962) revealed that GSTA4 expression was markedly elevated in malignant cells from ENZR tumors compared to those from non‐resistant counterparts (Figure 2B and Figure S2B–E). This finding was further validated by transcriptomic sequencing of ENZR cell lines (Figure S2F), with subsequent confirmation at both the mRNA and protein levels via qPCR (Figure S2G) and Western blot (Figure 2C), respectively. Next, we established two CRPC in vivo models, as previously described [29]: a spontaneous *Pten/Trp53*‐knockout model (Figure 2D) and an orthotopic model derived from *Pten/Trp53*‐deficient prostate organoids (Figure 2F). Both models were subjected to ENZ treatment as illustrated. Therapeutic response was assessed by comparing prostate tumor volumes before and after ENZ treatment. Tumors exhibiting regression were classified as ENZ‐sensitive, whereas those continuing to grow were considered resistant (Figure S2H,J). IHC analysis revealed marked GSTA4 upregulation in resistant tumors from each model (Figure 2E,G and Figure S2M,P). Furthermore, 4‐HNE staining indicated reduced lipid peroxidation in resistant tumors, which was negatively correlated with GSTA4 levels (Figure S2I,K,L,N,O,Q), suggesting a role of GSTA4 in redox regulation during treatment resistance.

In a clinical cohort of 158 PCa patients, GSTA4 expression was significantly elevated in tumors resistant to SGAT (Figure 2H,I). Elevated GSTA4 levels correlated with advanced tumor stage, increased prostate‐specific antigen (PSA) levels, and higher Gleason scores (Figure 2J–L). Kaplan‐Meier analysis revealed that high GSTA4 expression predicted reduced CRPC‐free survival (Figure 2M). Both univariate and multivariate Cox regression analyses confirmed GSTA4 as an independent prognostic factor (Figure 2N). Correlative analysis of MRI and IHC data from 8 paired pre‐ and post‐SGAT samples demonstrated that elevated GSTA4 expression was associated with a poorer treatment response (Figure 2O,P). Collectively, our multi‐level evidence identifies GSTA4 as a key ferroptosis suppressor driving ENZ resistance in CRPC, serving as an independent prognostic biomarker for SGAT response.

### 2.3 GSTA4 Governs ENZ Resistance by Regulating Lipid Peroxidation

**FIGURE 3:**
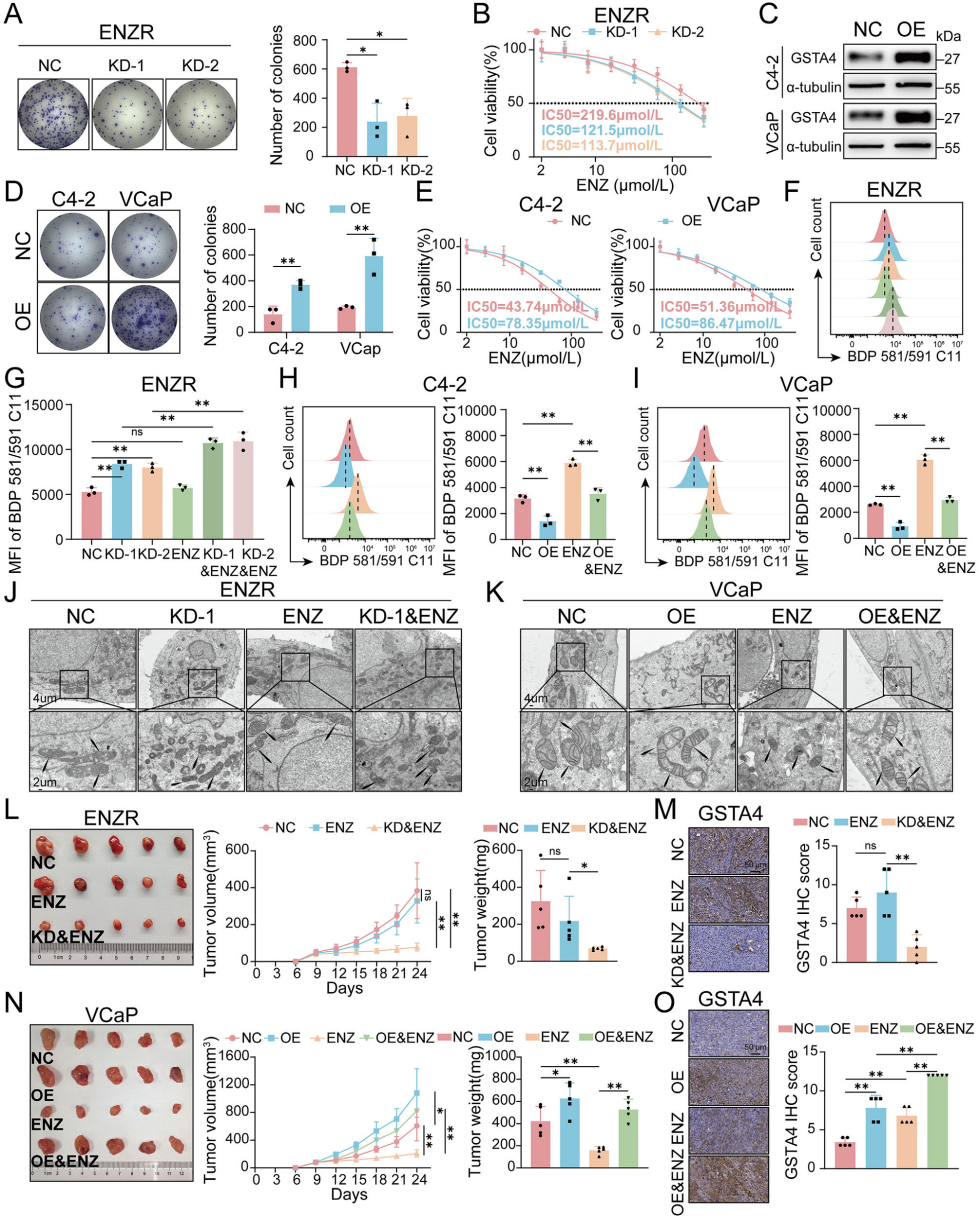
GSTA4 governs ENZ resistance by regulating lipid peroxidation. (A) Colony formation assays showed that GSTA4‐KD reduced colony formation in ENZR cells following ENZ treatment. (B) Cell viability assays demonstrated that GSTA4‐KD enhanced cellular sensitivity to ENZ in ENZR cells. (C) Western blot analysis confirmed the efficiency of GSTA4‐OE in wild‐type C4‐2 and VCaP cells. (D) Colony formation assays indicated that GSTA4‐OE conferred ENZ resistance in wild‐type cells. (E) Cell viability assays showed that GSTA4‐OE decreased cellular sensitivity to ENZ in both wild‐type C4‐2 and VCaP cells. (F, G) Lipid peroxidation assays revealed that GSTA4‐KD in combination with ENZ treatment significantly increased lipid peroxidation in ENZR cells. (H, I) Lipid peroxidation assays showed that GSTA4‐OE attenuated ENZ‐induced lipid peroxidation in wild‐type C4‐2 (H) and VCaP (I) cells. (J, K) TEM analysis revealed mitochondrial morphological features characteristic of ferroptosis in GSTA4‐KD ENZR cells under ENZ treatment, while GSTA4‐OE protected against these morphological changes in wild‐type VCaP cells. Scale bars: 4 µm (upper panels), 2 µm (lower panels). (L) GSTA4‐KD in combination with ENZ treatment potently inhibited tumor growth and reduced tumor weight in xenografts derived from ENZR cells. (M) Representative IHC staining images of GSTA4 expression in ENZR cell‐derived xenografts. Scale bar, 50 µm. (N) GSTA4‐OE abrogated the antitumor efficacy of ENZ, restoring tumor growth and weight in wild‐type xenografts. (O) Representative IHC staining images of GSTA4 in wild‐type xenografts with GSTA4‐OE and ENZ treatment. Scale bar, 50 µm.

To determine whether GSTA4 regulates lipid peroxidation and ferroptosis, we employed two independent shRNAs for GSTA4 knockdown (GSTA4‐KD) in C4‐2 and VCaP cells. Following ferroptosis induction with RSL3 or erastin, GSTA4‐KD potentiated the sensitivity of both cell lines to ferroptosis. This sensitization was selectively rescued by liproxstatin‐1 (Lip‐1) or ferrostatin‐1 (Fer‐1), but not by inhibitors of apoptosis (Z‐VAD), necrosis (Necro‐3), or autophagy (3‐MA) (Figure S3A,C). Following validation of efficient GSTA4 knockdown (GSTA4‐KD) by Western blot (Figure S3E), subsequent IC_50_ assays further validated that GSTA4‐KD enhanced cellular susceptibility to RSL3‐ or erastin‐induced ferroptosis, an effect abolished by Lip‐1, supporting a protective role of GSTA4 against ferroptosis (Figure S3B,D). In ENZR PCa cells, GSTA4‐KD significantly suppressed colony formation upon ENZ treatment and enhanced cellular sensitivity to ENZ, as evidenced by reduced viability and decreased IC_50_ values (Figure 3A,B). Conversely, GSTA4 overexpression (GSTA4‐OE) in wild‐type C4‐2 and VCaP cells, validated by Western blot (Figure 3C), promoted colony formation and conferred resistance to ENZ, as indicated by increased IC_50_ values under ENZ treatment (Figure 3D,E). Moreover, GSTA4 expression in wild‐type cells was upregulated by ENZ in a dose‐dependent manner (Figure S3F), suggesting a potential compensatory adaptive mechanism upon drug challenge.

Given the protective role of GSTA4 in ferroptosis, we next assessed lipid peroxidation, a key feature of ferroptosis and drug responsiveness. In ENZR cells, GSTA4‐KD in combination with ENZ treatment potently enhanced lipid peroxidation (Figure 3F,G). Conversely, GSTA4‐OE in wild‐type cells attenuated ENZ‐induced lipid peroxidation (Figure 3H,I). Fluorescence imaging further corroborated these findings, showing that GSTA4‐KD plus ENZ increased oxidized (green) BODIPY fluorescence in ENZR cells, indicating elevated peroxidation, while GSTA4‐OE diminished the ENZ‐triggered green signal and augmented red fluorescence, confirming a reduction in lipid peroxidation (Figure S3G,H). Consistent with these findings, transmission electron microscopy (TEM) revealed morphological features consistent with ferroptosis, including mitochondrial condensation and loss of cristae in GSTA4‐KD ENZR cells (Figure 3J), whereas TEM of GSTA4‐OE cells showed protection against ENZ‐induced mitochondrial damage (Figure 3K).

In vivo, GSTA4‐KD acted synergistically with ENZ to potently suppress tumor growth and reduce tumor weight in xenografts derived from ENZR cells (Figure 3L). Conversely, GSTA4‐OE abolished the antitumor efficacy of ENZ in wild‐type xenografts, restoring tumor growth and weight (Figure 3N). Notably, no significant differences in body weight were observed among the treatment groups in either xenograft model, indicating good treatment tolerability (Figure S3I,J). IHC analysis confirmed corresponding alterations in GSTA4 and 4‐HNE expression across the above in vivo models (Figure 3M,O and Figure S3K,L). Together, these findings demonstrate that GSTA4 modulates ENZ resistance in PCa by regulating lipid peroxidation both in vitro and in vivo, highlighting its potential as a therapeutic target to overcome resistance to ENZ.

### 2.4 GSTA4 Catalytic Activity Enables Unconventional Mechanisms to Protect Against Ferroptosis

**FIGURE 4:**
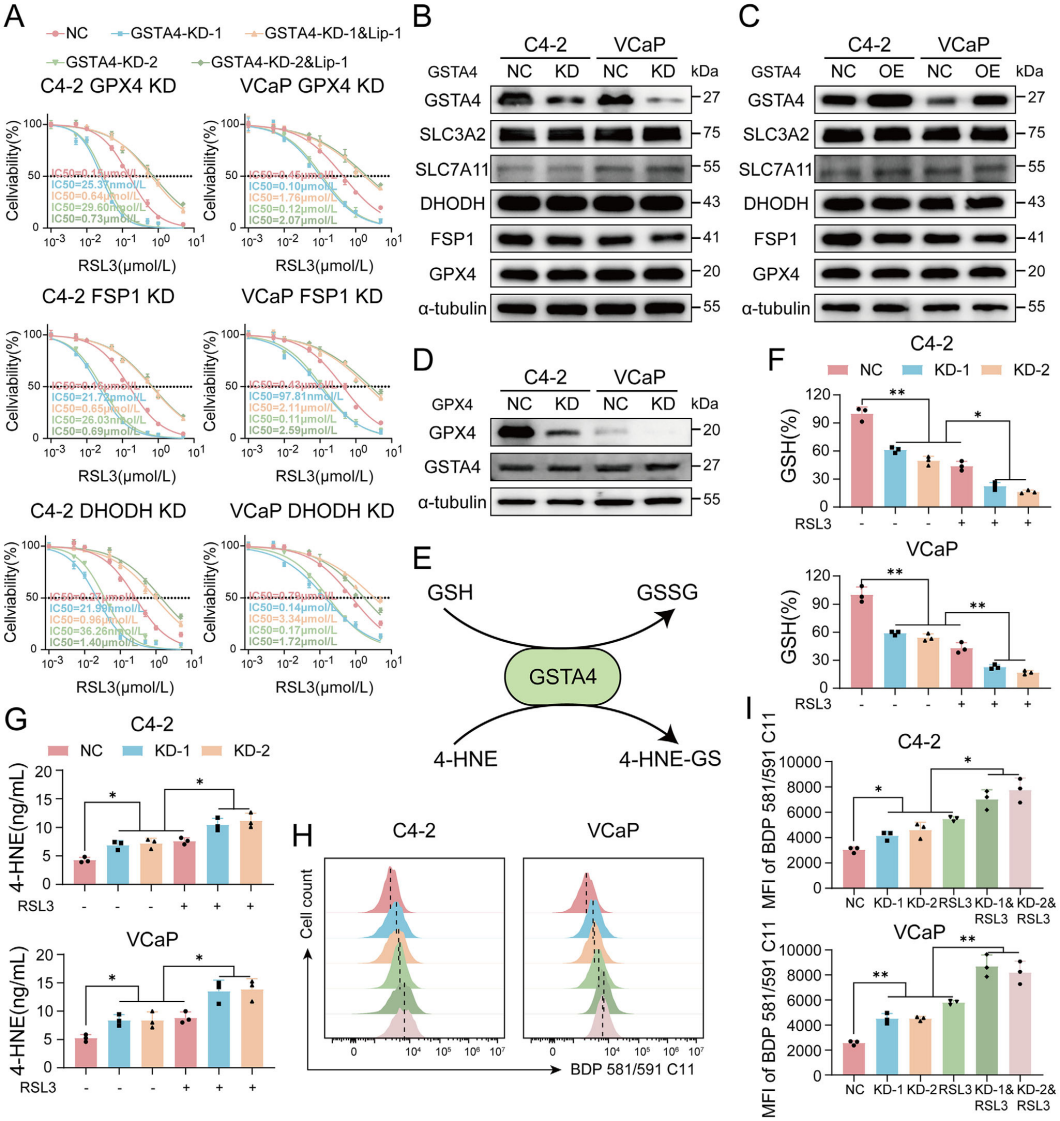
GSTA4 catalytic activity powers unconventional mechanisms to protect against ferroptosis. (A) Cell viability assays of C4‐2 and VCaP cells with GSTA4‐KD plus Lip‐1 treatment after RSL3 stimulation upon knockdown of GPX4, FSP1, or DHODH. (B, C) Western blot analysis of GSTA4, SLC3A2, SLC7A11, DHODH, FSP1, and GPX4 in NC and GSTA4‐KD (B) and GSTA4‐OE cells (C). (D) Western blot analysis of GSTA4 protein levels in C4‐2 and VCaP cells upon GPX4‐KD. (E) Schematic illustration of GSTA4‐mediated detoxification: GSTA4 conjugates GSH to 4‐HNE to form 4‐HNE‐GS and participates in GSH/GSSG cycling. (F, G) Assays of GSH (F) and 4‐HNE (G) levels in NC and GSTA4‐KD C4‐2 and VCaP cells with or without RSL3 treatment. (H, I) Flow cytometry analysis (H) and quantification (I) of lipid peroxidation in NC and GSTA4‐KD C4‐2 and VCaP cells treated with RSL3.

Given the critical role of GSTA4 in maintaining cellular redox homeostasis [24], we sought to examine the underlying mechanism by which its catalytic activity confers resistance to ferroptosis. We first examined the relationship between GSTA4 and key established ferroptosis defense systems, including the canonical GPX4 axis and the compensatory pathways involving FSP1 and DHODH. Notably, GSTA4 silencing remained capable of sensitizing cells to RSL3 or erastin even following the individual knockdown of GPX4, FSP1, or DHODH (Figure 4A and Figure S4A). Western blot analysis indicated neither GSTA4‐KD (Figure 4B) nor GSTA4‐OE (Figure 4C) affected the protein expression of SLC3A2, SLC7A11, DHODH, FSP1, or GPX4. Conversely, GSTA4 expression remained unchanged upon the knockdown of GPX4, FSP1, or DHODH (Figure 4D and Figure S4B,C), suggesting that GSTA4 functions independently of these canonical pathways. Based on these findings, we next investigated its catalytic function in conjugating GSH to 4‐HNE to generate 4‐HNE‐GS and promoting GSH/GSSG cycling (Figure 4E). GSTA4‐KD significantly reduced cellular GSH levels while enhancing 4‐HNE accumulation, both under basal conditions and upon RSL3 challenge (Figure 4F,G). In line with these metabolic alterations, flow cytometry confirmed that GSTA4‐KD markedly augmented RSL3‐induced lipid peroxidation (Figure 4H,I). Taken together, these results demonstrate that GSTA4 confers ferroptosis resistance primarily through its catalytic activity in detoxifying 4‐HNE and maintaining intracellular GSH levels, independent of classical ferroptosis regulatory mechanisms.

### 2.5 Mitochondrial Translocation Enables GSTA4 to Interact With PGAM5 and Suppress Ferroptosis Independently of GSH

**FIGURE 5:**
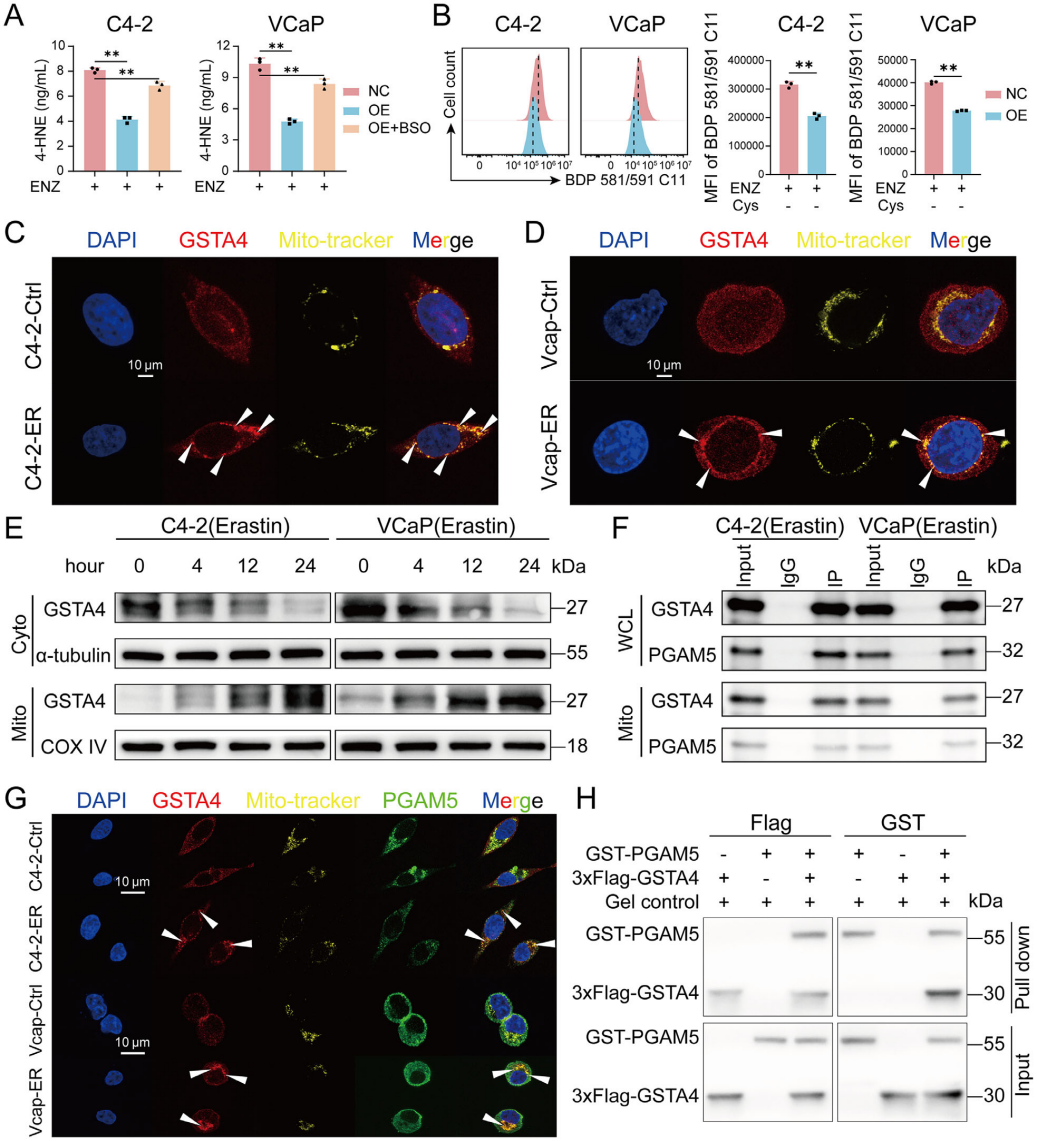
Mitochondrial translocation enables GSTA4 to interact with PGAM5 and suppress ferroptosis independently of GSH. (A) 4‐HNE levels in C4‐2 and VCaP cells under erastin‐induced stress, comparing control (NC), GSTA4‐OE, and GSTA4‐OE cells treated with BSO. (B) Flow cytometric analysis of lipid peroxidation in C4‐2 and VCaP cells (NC vs. OE) cultured in cystine‐free medium with erastin. (C, D) Confocal immunofluorescence images of C4‐2 (C) and VCaP (D) cells demonstrating GSTA4 translocation to mitochondria upon erastin treatment. Scale bar, 10 µm. (E) Western blot analysis of GSTA4 expression in cytoplasmic and mitochondrial fractions from C4‐2 and VCaP cells over a time course of erastin treatment. (F) Co‐IP analysis of GSTA4 and PGAM5 in whole cell lysates (WCL) and mitochondrial fractions of C4‐2 and VCaP cells under erastin treatment. (G) Confocal microscopy images showing colocalization of GSTA4 (red), PGAM5 (green), and mitochondria (MitoTracker, yellow) in C4‐2 and VCaP cells under erastin stress. Scale bar, 10 µm. (H) GST pull‐down assay confirms the direct binding between GSTA4 and PGAM5.

To determine whether the anti‐ferroptotic function of GSTA4 depends on GSH availability, we performed rescue experiments with GSTA4‐OE under conditions of complete GSH depletion. Notably, enforced expression of GSTA4 effectively reversed ENZ‐induced 4‐HNE accumulation even under complete GSH synthesis inhibition by buthionine sulfoximine (BSO), a potent inhibitor of γ‐glutamylcysteine synthetase (Figure 5A). Moreover, GSTA4‐OE significantly attenuated ENZ‐triggered lipid peroxidation even under complete GSH depletion in cystine‐free medium (Figure 5B). Together, these findings demonstrate that GSTA4 mediates a GSH‐independent detoxification program that alleviates lipid peroxidation and ferroptosis. To elucidate the underlying mechanism, we analyzed scRNA‐seq data (Figure S2B–E) and observed that biological processes related to mitochondrial function and redox homeostasis were significantly enriched in GSTA4‐high cells (Figure S5A). This prompted us to hypothesize that GSTA4 may localize to mitochondria to exert its protective effects. Upon erastin treatment, which inhibits SLC7A11‐mediated cystine uptake and thereby limits GSH synthesis [30], confocal imaging (Figure 5C,D) and cellular fractionation followed by Western blot analysis (Figure 5E) consistently revealed a time‐dependent translocation of GSTA4 from the cytoplasm to the mitochondria. Furthermore, TEM analysis showed that GSTA4‐OE alleviated ferroptosis‐associated mitochondrial damage (Figure 3K), supporting a protective role of mitochondrial GSTA4 during ferroptotic stress.

To elucidate how mitochondrial translocation of GSTA4 inhibits ferroptosis, we performed mass spectrometry to identify GSTA4‐interacting proteins under erastin treatment. Among the top 20 candidates, PGAM5 emerged as a prominent mitochondria‐associated interactor (Figure S5B,C) and has been reported as a mitochondrial phosphatase involved in regulating cell death and metabolic homeostasis [31, 32]. Molecular docking further predicted the structural basis of the GSTA4‐PGAM5 interaction (Figure S5D). This interaction was validated by co‐IP in both whole‐cell and mitochondrial fractions upon erastin treatment (Figure 5F), and confocal microscopy revealed clear mitochondrial colocalization of GSTA4 and PGAM5 under erastin treatment (Figure 5G), confirming PGAM5 as a mitochondrial interaction partner of GSTA4. Importantly, a GST pull‐down assay verified direct binding between GSTA4 and PGAM5 (Figure 5H). Collectively, these data demonstrate that GSTA4 translocates to mitochondria and directly interacts with PGAM5, thereby modulating redox homeostasis, preserving mitochondrial integrity, and ultimately inhibiting ferroptosis under oxidative stress through GSH‐independent mechanisms.

### 2.6 Mapping the GSTA4‐PGAM5 Interaction Domain Essential for Regulating Mitochondrial Drp1 Dephosphorylation

**FIGURE 6:**
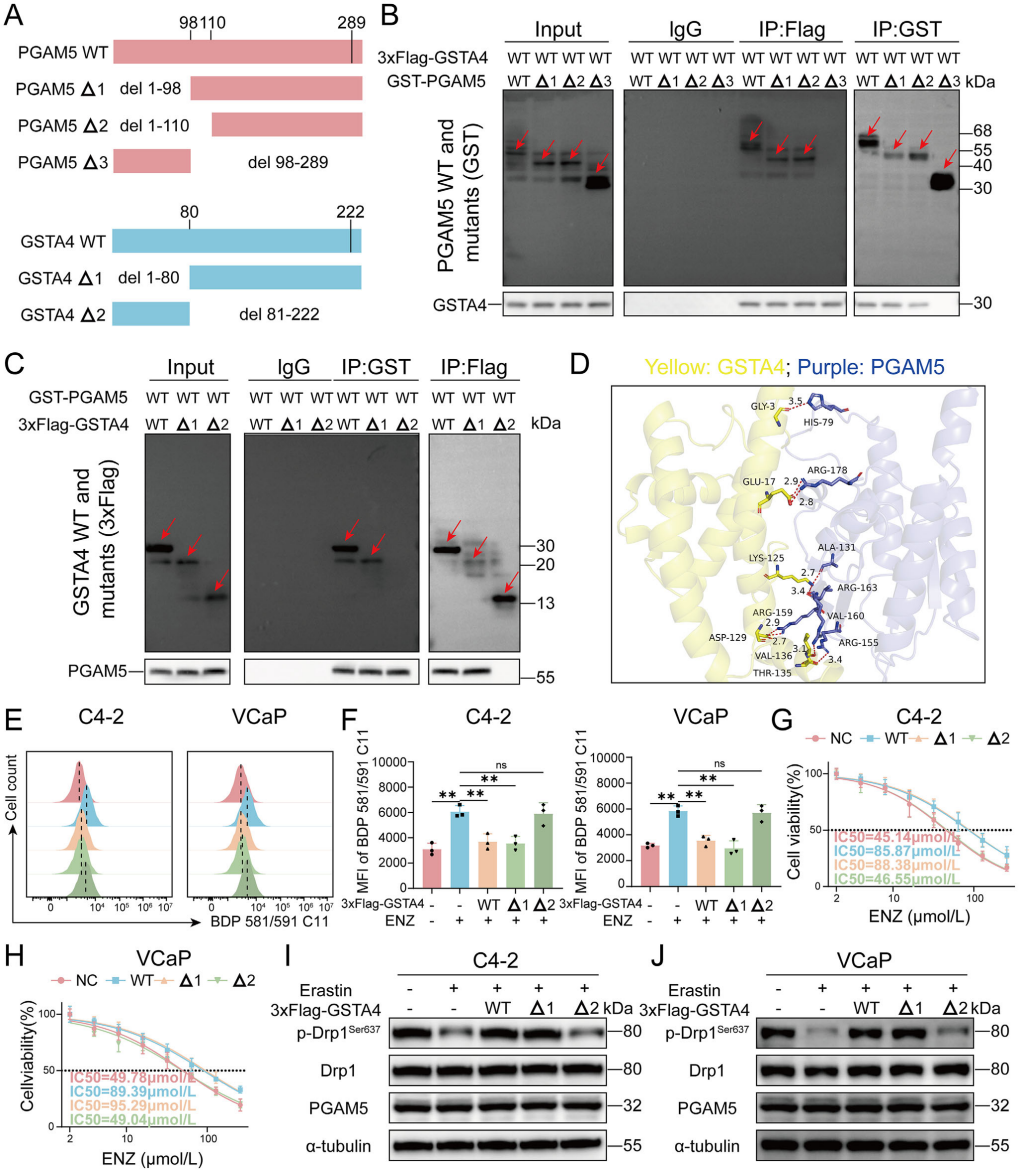
Mapping the GSTA4‐PGAM5 interaction domain essential for regulating mitochondrial Drp1 dephosphorylation. (A) Schematic illustrations of full‐length and truncated constructs of PGAM5 and GSTA4 used for interaction mapping. (B,C) Co‐IP assays in HEK293T cells co‐expressing 3xFlag‐GSTA4 and GST‐PGAM5 variants. Truncated mutants were used to identify essential interaction regions. (D) Predicted molecular docking model of the GSTA4 Δ1 and PGAM5 Δ1 complex. (E, F) Flow cytometry analysis measuring lipid peroxidation in C4‐2 and VCaP cells expressing GSTA4 WT, Δ1, or Δ2, with or without ENZ treatment. (G, H) Cell viability assays showing ENZ IC_50_ values in C4‐2 (G) and VCaP (H) cells, including NC and cells expressing GSTA4 WT, Δ1, or Δ2. (I, J) Western blot analysis of phospho‐Drp1(Ser637), Drp1, and PGAM5 in C4‐2 (I) and VCaP (J) cells expressing GSTA4 WT, Δ1, or Δ2 under erastin treatment.

To delineate the critical interaction domain between GSTA4 and PGAM5, we generated a series of truncated constructs of both proteins (Figure 6A). Based on previous reports [33, 34], PGAM5 was divided into three fragments (amino acids 1–98, 98–289, and 110–289), whereas GSTA4 was divided into two fragments (amino acids 1–80 and 81–222) according to its domain architecture in UniProt. The N‐terminal fragment (1–80 aa) contains the glutathione‐binding site (G‐site), whereas the C‐terminal fragment (81–222 aa) encompasses the hydrophobic substrate‐binding site (H‐site) responsible for substrate recognition. Co‐IP assays in HEK293T cells co‐expressing full‐length or truncated versions of 3xFlag‐GSTA4 and GST‐PGAM5 revealed that their binding is primarily mediated by residues 81–222 of GSTA4 and 98–289 of PGAM5 (Figure 6B,C). Molecular docking analysis further predicted that the interaction between the GSTA4 (81‐222) and PGAM5 (98‐289) fragments involves more binding sites and shorter hydrogen bond lengths than that between the full‐length proteins, suggesting a potentially favorable binding interface (Figure 6D).

Functionally, both GSTA4‐WT and the Δ1 mutant significantly attenuated ENZ‐enhanced lipid peroxidation in C4‐2 and VCaP cells, whereas the Δ2 mutant was ineffective (Figure 6E,F). Consistent with these findings, cell viability assays demonstrated that GSTA4 WT or Δ1 increased the IC50 of ENZ in both cell lines, whereas the Δ2 mutant failed to confer ENZ resistance (Figure 6G,H). Previous studies have shown that PGAM5 regulates Drp1 dephosphorylation in mitochondria, thereby activating and promoting its mitochondrial translocation, which drives mitochondrial fission and accelerates lipid peroxidation during ferroptosis [35]. Western blot analysis indicated that erastin‐induced oxidative stress promoted Drp1 dephosphorylation at Ser637 [36] (Figure 6I,J). Accordingly, GSTA4 WT or Δ1 mutant preserved phospho‐Drp1 (Ser637) levels upon erastin treatment without altering PGAM5 expression in both cell lines (Figure 6I,J), whereas the GSTA4 Δ2 mutant failed to prevent erastin‐induced Drp1 dephosphorylation (Figure 6I,J). Consistently, confocal microscopy showed that GSTA4 WT and Δ1 mutant, but not the Δ2 mutant, inhibited erastin‐induced mitochondrial translocation of Drp1 in C4‐2 (Figure S6A) and VCaP (Figure S6B) cells. Collectively, these results demonstrate that a specific GSTA4–PGAM5 interaction, mediated by the GSTA4 (81–222) and PGAM5 (98–289) domains, restrains erastin‐induced Drp1 dephosphorylation at Ser637, thereby limiting Drp1 mitochondrial translocation, mitochondrial dysfunction, lipid peroxidation, and reducing ENZ sensitivity.

### 2.7 Targeting GSTA4 With Metformin Restores Ferroptosis and Overcomes ENZ Resistance

**FIGURE 7:**
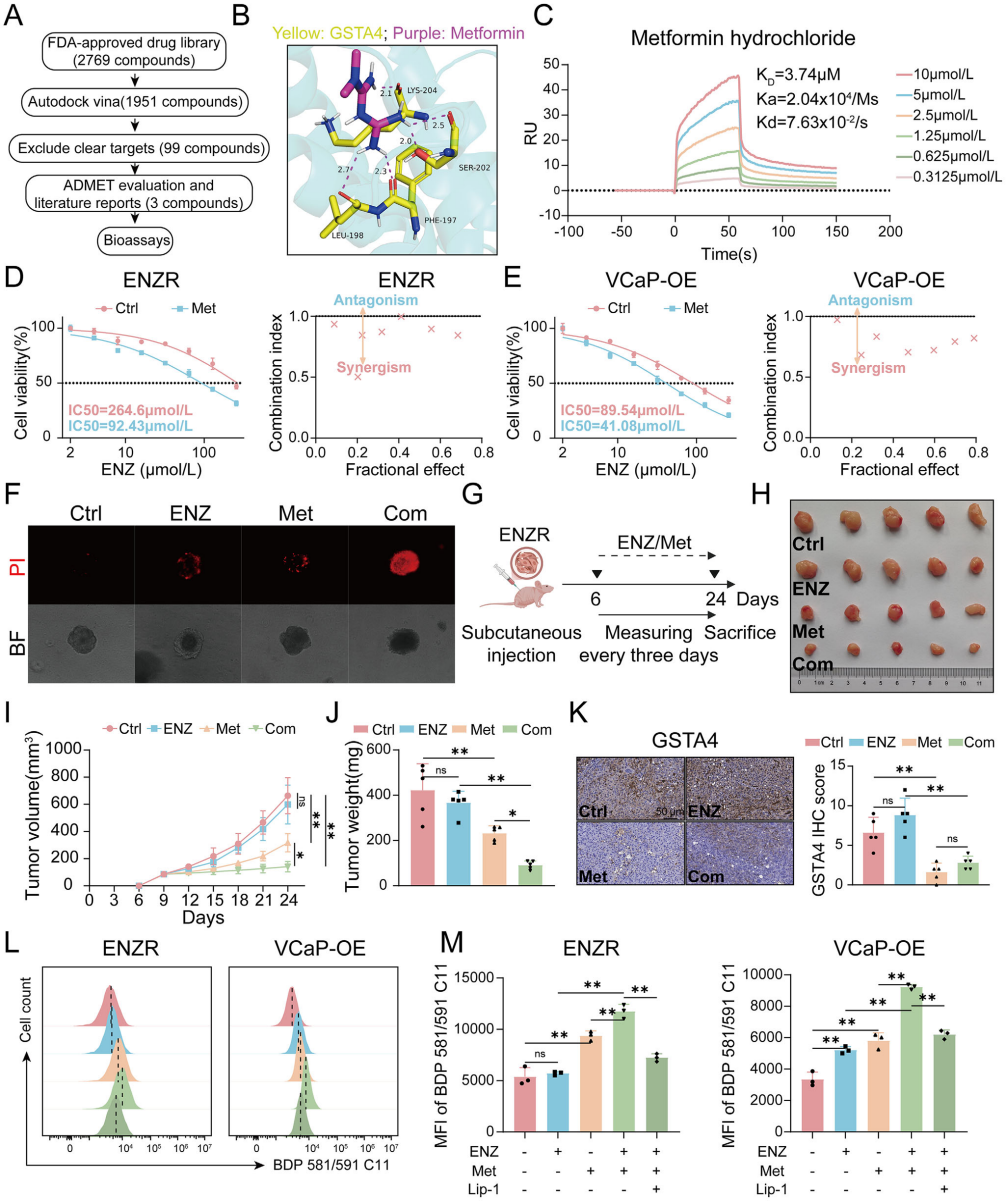
Targeting GSTA4 with metformin restores ferroptosis and overcomes ENZ resistance. (A) Workflow of virtual screening for FDA‐approved drugs against GSTA4. (B) Molecular docking model of GSTA4 (yellow) with metformin (purple). (C) SPR assay characterizing the binding of metformin hydrochloride to GSTA4, with a dissociation constant (K_D_ = 3.74 µM). (D, E) Cell viability and CI analysis in ENZR (D) and VCaP‐OE (E) cells treated with ENZ and/or Met. IC_50_ values are indicated, and a CI value < 1 denotes synergism. (F) PI‐stained (top, red) and bright‐field (BF, bottom) images of *Pten/Trp53*‐deficient PCa organoids treated with ENZ and/or Met. (G–J) In vivo xenograft assay using ENZR cells. Schematic of the experimental design (G), representative images of resected tumors from each group (H), tumor volume kinetics over 24 days (I), and tumor weight at sacrifice (J). (K) IHC staining of GSTA4 in xenograft tumors (left) and corresponding quantification of IHC scores (right). Scale bar: 50 µm. (L, M) Flow cytometry analysis (L) and quantification (M) of lipid peroxidation in ENZR and VCaP‐OE cells treated as indicated with or without Lip‐1.

To tackle the clinical challenge of ENZ resistance, we conducted virtual screening of an FDA‐approved drug library (2,769 compounds) against GSTA4 using AutoDock Vina, yielding 1,951 candidate compounds. After excluding compounds with previously reported direct links to AR signaling or ferroptosis‐related targets, we performed ADMET profiling covering drug‐likeness, toxicity, and pharmacokinetic properties, followed by literature evaluation of preclinical relevance. This stepwise screening yielded 3 candidate compounds: atorvastatin, celecoxib, and metformin (Figure 7A). Functional validation in ENZR cells revealed that only metformin showed a markedly reduced anti‐proliferative effect in GSTA4‐KD ENZR cells, whereas its activity was enhanced in GSTA4‐OE VCaP cells, indicating a GSTA4‐dependent mechanism (Figure S7A,B). Molecular docking predicted that metformin occupies the putative active‐site pocket of GSTA4 (Figure 7B), suggesting a structural basis for its targeted action. Molecular dynamics simulations further confirmed a stable binding mode between metformin and GSTA4, as indicated by consistent RMSD, radius of gyration, RMSF, and SASA profiles (Figure S7C–F). Western blot analyses consistently demonstrated elevated GSTA4 expression in ENZR versus ENZ‐sensitive (Sen) cells, which was significantly reduced upon metformin treatment (Figure S7G). Moreover, metformin reduced GSTA4 protein abundance in a concentration‐dependent manner in C4‐2 and VCaP cells (Figure S7H). To determine whether this effect depends on canonical AMPK signaling, cells were co‐treated with the AMPK inhibitor dorsomorphin. Although dorsomorphin effectively blocked metformin‐induced AMPK phosphorylation, metformin‐mediated downregulation of GSTA4 was largely preserved in both C4‐2 and VCaP cells, indicating that this effect was largely independent of the canonical AMPK pathway (Figure S7I). Additionally, surface plasmon resonance (SPR) assays confirmed specific and high‐affinity binding between metformin and GSTA4 (Figure 7C).

We next assessed the combined effect of metformin and ENZ in vitro and in vivo. Metformin synergized with ENZ to suppress viability in ENZR and VCaP‐OE cells, as evidenced by reduced IC_50_ values and a combination index (CI) below 1 (Figure 7D,E). Colony formation assays further confirmed a marked reduction in colony number under combination treatment in both ENZR and VCaP‐OE cells (Figure S7J,K). Enhanced cell death was also observed in *Pten/Trp53*‐deficient PCa organoids stained with propidium iodide (PI) following combination treatment (Figure 7F), corroborating the in vitro viability findings. In vivo, a subcutaneous xenograft model established with ENZR cells (Figure 7G) showed that tumors from the combination treatment group exhibited markedly reduced volume (Figure 7I) and weight (Figure 7J), as visualized upon resection (Figure 7H). Importantly, no significant differences in body weight were observed among the treatment groups throughout the treatment period, indicating that the combination therapy was well tolerated in vivo (Figure S7L). IHC analysis further indicated altered expression of GSTA4 and 4‐HNE in xenograft tumors (Figure 7K and Figure S7M). Given the central role of lipid peroxidation in ferroptosis, flow cytometry analysis demonstrated that combined metformin and ENZ treatment significantly increased lipid peroxidation in ENZR and VCaP‐OE cells, and these effects were reversed by Lip‐1 (Figure 7L,M). Collectively, these results indicate that metformin overcomes ENZ resistance in CRPC by targeting GSTA4 to promote ferroptosis, an effect mediated through enhanced lipid peroxidation.

## 3 Discussion

The emergence of therapeutic resistance in cancer frequently results from the activation of compensatory survival pathways [15, 37, 38]. In this study, we demonstrate that ferroptosis suppression accompanied by reduced lipid peroxidation represents a hallmark of ENZ resistance and that GSTA4 is associated with adverse clinical outcomes. A central feature of this adaptive response is a spatially coordinated defense network orchestrated by GSTA4 in ENZR CRPC. This network effectively suppresses ferroptosis, a process tightly linked to therapy sensitivity [39, 40, 41, 42], by harmonizing antioxidant responses across two distinct cellular compartments: GSH‐dependent defense in the cytosol and GSH‐independent protection in the mitochondria. These findings reveal a previously unrecognized mechanism of treatment resistance and show that cancer cells repurpose GSTA4 to establish a multi‐compartmental defense system under therapeutic pressure.

GSH‐dependent pathways are fundamental to neutralizing lipid peroxides, primarily through the activity of enzymes such as GPXs and GSTs [43, 44]. While GPX4 and parallel compensatory systems FSP1 and DHODH are well‐established regulators of ferroptosis [45, 46, 47], the contribution of specific GST isoforms remains less defined. An important finding of this study is a non‐canonical ferroptosis‐suppressive pathway mediated by GSTA4, which confers robust protection against ferroptosis independently of the canonical GPX4 system and other known compensatory defense pathways such as FSP1 or DHODH, via direct detoxification of 4‐HNE [46, 47]. Notably, we uncovered a stress‐induced translocation of GSTA4 to the mitochondria, where it interacts with the phosphatase PGAM5, stabilizes inhibitory phosphorylation of Drp1 at Ser637, and prevents excessive mitochondrial fission [35, 36]. This finding fundamentally expands the functional repertoire of GSTA4 from traditional antioxidant metabolism to the direct regulation of mitochondrial dynamics. Given that damaged mitochondria are both a source and a target of lipid peroxidation, this non‐canonical, scaffolding function of GSTA4 adds a crucial line of defense against ferroptosis by preserving mitochondrial network integrity. Consistent with this mechanism, the perinuclear accumulation of mitochondria observed in our study likely reflects stress‐induced mitochondrial remodeling and redistribution under oxidative stress. Our findings place a member of the GST enzyme family at a central position in mitochondrial ferroptosis regulation and provide a specific molecular link to the growing recognition of mitochondrial involvement in ferroptosis. However, the relative contributions of the enzymatic and scaffold functions of GSTA4 to therapy resistance, as well as potential shifts in this balance during disease progression, remain open questions for further investigation.

In this study, the computational screening and subsequent experimental validation identified metformin as a GSTA4‐binding agent that disrupts this resistance axis and supports opportunities for drug repurposing. Our findings suggest that targeting GSTA4 represents a viable strategy to overcome ENZ resistance. Nevertheless, metformin exerts pleiotropic biological effects and its in vivo antitumor activity may not solely reflect GSTA4 inhibition [48, 49]. Future research should prioritize the development of selective GSTA4 inhibitors and the evaluation of the therapeutic potential of GSTA4‐directed interventions, while assessing GSTA4 expression as a predictive biomarker to enable rational patient stratification for combination therapies incorporating ENZ and metformin or more selective inhibitors, thereby advancing precision treatment strategies for CRPC.

In conclusion, we propose a model in which GSTA4 functions as a key regulator of the defense against ferroptosis in CRPC through a dual‐compartmental mechanism. This work broadens the current framework of non‐canonical ferroptosis regulation and highlights GSTA4 as a promising therapeutic target.

## 4 Methods

### 4.1 Cell Culture

The human CRPC cell lines (C4‐2 and VCaP) and the human embryonic kidney cell line (HEK293T) were acquired from the American Type Culture Collection. C4‐2‐ENZR subline (C4‐2‐ENZR) was established via progressive adaptation to ENZ, with concentrations increasing from 10 to 30 µM, following a previously reported protocol [9]. All cells were maintained at 37°C under 5% CO_2_ in a humidified atmosphere. C4‐2, VCaP, and C4‐2‐ENZR were cultured in RPMI‐1640 medium (Gibco) supplemented with 10% fetal bovine serum (FBS, NEWZERUM) and 1% penicillin‐streptomycin (Gibco). HEK293T cells were grown in DMEM (Gibco) containing 10% FBS and 1% antibiotics. To maintain resistance, 10  µM ENZ was consistently added to the culture medium of C4‐2‐ENZR cells. All cell lines were routinely authenticated by STR profiling and confirmed to be mycoplasma‐free.

### 4.2 Organoids

PCa organoids were established from *Pten/Trp53* double‐knockout mice exhibiting spontaneous prostate adenocarcinoma. For organoid generation, isolated tumor cells were suspended in Matrigel (Corning) and plated in 24‐well plates at a density of 5,000 cells per well [29]. Cultures were maintained in advanced prostate organoid medium under standard conditions and routinely propagated upon reaching 70%–80% confluence.

### 4.3 Mice

All animal procedures were approved by the Institutional Animal Care and Use Committee of Sun Yat‐sen Memorial Hospital, Sun Yat‐sen University. *Pten/Trp53* double‐knockout mice, which develop spontaneous prostate adenocarcinoma, were subjected to androgen deprivation therapy (ADT) by surgical castration, followed by ENZ treatment (Selleck, 10 mg/kg, 3 times/week) to induce ENZR CRPC. For orthotopic organoid transplantation, PCa organoids derived from *Pten/Trp53*‐deficient mice were collected and inoculated into the prostates of 8‐week‐old C57BL/6J mice (3 organoid cultures per mouse, equivalent to one confluent 48‐well plate culture). Tumors were allowed to develop for 12 weeks before ADT was administered to induce ENZR CRPC. For subcutaneous xenografts, 5 × 10^6^ cells of C4‐2‐ENZR, VCaP, or their derivatives were inoculated into the flanks of BALB/c nude mice. Treatments with ENZ (10 mg/kg, 3 times/week), metformin (250 mg/kg/day, MCE), or ferroptosis inhibitors (Selleck, Lip‐1, 10 mg/kg/day; Fer‐1, 5 mg/kg/day) commenced 9 days post‐injection when tumors reached 50–100 mm^3^. Tumor volume was monitored every three days and calculated as V = length × width^2^ × 0.5. Mice were euthanized at defined endpoints or when tumor burden exceeded IACUC guidelines. The animal study was performed with the permission of the Animal Care and Use Committee of Sun Yat‐Sen University.

### 4.4 Patient Samples

Formalin‐fixed, paraffin‐embedded (FFPE) tissue sections from 158 clinically diagnosed PCa patients were obtained from Sun Yat‐sen Memorial Hospital, Sun Yat‐sen University. Comprehensive clinical and therapeutic data were collected for all cases. The baseline clinicopathological characteristics of the patient cohort are summarized in Table S1. Among these, eight patients had paired pre‐ and post‐treatment magnetic resonance imaging (MRI) scans available for correlative analysis. The use of human specimens was approved by the Institutional Review Board of Sun Yat‐sen Memorial Hospital and informed consent was obtained from all participants.

### 4.5 Plasmids and Cell Transfection

For GSTA4 OE, the full‐length human GSTA4 cDNA was cloned into a pCDH‐CMV vector (IGE Biotechnology). Wild‐type C4‐2 and VCaP cells were transfected using X‐tremeGENE HP (Roche) according to the manufacturer's protocol and selected with puromycin to establish stable overexpression lines. For stable knockdown experiments, lentiviral vectors expressing shRNA targeting GSTA4, GPX4, FSP1, or DHODH (IGE Biotechnology) were packaged in HEK293T cells using psPAX2 and pMD2.G. Supernatants were collected, filtered, and used to transduce C4‐2‐ENZR, wild‐type C4‐2, and VCaP cells. Transduced cells were selected with puromycin to generate stable knockdown pools for subsequent in vitro and in vivo studies. The corresponding shRNA sequences are listed in Table S2.

### 4.6 Cell Viability Assay

Cell viability was assessed using the CCK‐8 assay (ApexBio). C4‐2‐ENZR, C4‐2, and VCaP cells were seeded in 96‐well plates and treated with ENZ, Lip‐1, Fer‐1, RSL3 (Selleck), erastin (Selleck), metformin, or various cell death inhibitors (MCE) at indicated concentrations. After treatment, 10 µL of CCK‐8 reagent was added to each well, and the absorbance was measured at 450 nm. Drug combination effects were evaluated using CalcuSyn software, with combination index (CI) values < 1, = 1, and > 1 indicating synergy, additivity, and antagonism, respectively. For colony formation assays, 1,000 cells per well were seeded in 6‐well plates and treated as specified for two weeks. Colonies were fixed with 4% paraformaldehyde, stained with 0.1% crystal violet, and quantified using image analysis software.

### 4.7 BODIPY 581/591 C11 Lipid Peroxidation Assay

C4‐2‐ENZR, C4‐2, and VCaP cells were seeded in 6‐well plates and treated with ENZ, ferroptosis inhibitors, RSL3, or metformin. To assess lipid peroxidation, cells were stained with BODIPY 581/591 C11 (DOJINDO, L267) at 37°C for 30 min, washed with PBS, and analyzed by flow cytometry (Beckman CytoFLEX) or confocal microscopy (Leica SP8 STED 3X). In addition, the corresponding quantitative analyses were performed using median fluorescence intensity (MFI).

### 4.8 PI Staining

For assessment of cell death in organoids, PCa organoids embedded in Matrigel were cultured in 48‐well plates and treated with ENZ and metformin. Following treatment, organoids were stained with propidium iodide (PI) (Elabscience, E‐CK‐A211) for 15 min and visualized under a fluorescence microscope.

### 4.9 GSH and 4‐HNE Assay

After the indicated treatments in 6‐well plates, intracellular GSH and 4‐HNE levels were quantified using commercial assay kits. GSH content was measured with a Glutathione Assay Kit (Beyotime, S0052) by detecting absorbance at 412 nm. 4‐HNE levels were determined using a 4‐HNE ELISA kit (Elabscience, E‐EL‐0128) according to the manufacturer's instructions, with absorbance read at 450 nm. All measurements were normalized to total protein concentration.

### 4.10 Immunohistochemistry Staining

FFPE tissue specimens from PCa patients, along with xenograft tumor samples from animal studies, were sectioned at 5 µm thickness (Servicebio). After deparaffinization, rehydration, and antigen retrieval, sections were incubated with primary antibodies followed by appropriate secondary antibodies (zsbio, PV‐6000) using a standard IHC detection system [50]. Immunostaining was evaluated by two independent pathologists using a semiquantitative scoring system based on staining intensity (0‐3) and percentage of positive cells (0‐4). The final immunoreactivity score (IRS) was calculated by multiplying these two values, yielding a range from 0 to 12.

### 4.11 Western Blot and qRT‐PCR

Whole‐cell lysates were extracted using RIPA buffer (Beyotime, P0013B) supplemented with protease (MCE, HY‐K0010) and phosphatase (Servicebio, G2007) inhibitors. Mitochondrial fractions were isolated using a Mitochondria Isolation Kit (Solarbio, SM0020) according to the manufacturer's instructions. Protein samples were separated by SDS‐PAGE (Servicebio, G2175, G2177), transferred to PVDF membranes (Merck Millipore, IPVH00010), and incubated with primary antibodies (GSTA4, 17271‐1‐AP; PGAM5, 68116‐1‐Ig; SLC7A11, 26864‐1‐AP; DHODH, 14877‐1‐AP; FSP1, 20886‐1‐AP, Proteintech; GPX4, YM8430, Immunoway; Drp1, 8570, Phospho‐Drp1 (Ser637), 4867, Cell Signaling Technology) at 4 °C overnight. After incubation with HRP‐conjugated secondary antibodies (ABclonal, AS003, AS014), protein bands were visualized using an enhanced chemiluminescence detection system. α‐tubulin (66031‐1‐Ig, Proteintech) and COX IV (11242‐1‐AP, Proteintech) served as loading controls for whole‐cell and mitochondrial fractions, respectively. Total RNA was extracted from cells using TRIzol reagent (Servicebio, G3013) and reverse‐transcribed into cDNA with HiScript II RT SuperMix (Vazyme, R223‐01). Quantitative real‐time PCR was performed using ChamQ Universal SYBR qPCR Master Mix (GOONIE, 500‐102) on a QuantStudio DX Real‐Time PCR System (ABI Quanstudio DX). The expression of target genes was normalized to GAPDH, and relative quantification was calculated using the 2^−ΔΔCt^ method. Primer sequences are listed in Table S2.

### 4.12 Transmission Electron Microscopy (TEM)

C4‐2‐ENZR and VCaP cells were fixed with 2.5% glutaraldehyde (Servicebio, G1102) in PBS at 4 °C for 2 h, followed by post‐fixation in 1% osmium tetroxide. After dehydration through a graded acetone series, the samples were embedded in epoxy resin. Ultrathin sections (70 nm) were cut and stained with uranyl acetate and lead citrate. Mitochondrial ultrastructure was examined using a transmission electron microscope operated at 80 kV.

### 4.13 Immunofluorescence and Colocalization Analysis

C4‐2 and VCaP cells were seeded at a density of 2 × 10^4^ cells per well in confocal dishes. For mitochondrial labeling, cells were incubated with MitoTracker (Yeasen, 40741ES50) for 40 min at 37°C prior to fixation. Cells were fixed with 4% paraformaldehyde for 20 min, permeabilized with 0.3% Triton X‐100 for 20 min, and blocked with 3% BSA for 30 min. After incubation with primary antibodies at 4°C overnight, samples were washed and stained with fluorophore‐conjugated secondary antibodies (ApexBio, K1204, K1212) for 30 min at room temperature. Nuclei were counterstained with DAPI (Servicebio, G1012). Images were acquired using a confocal microscope and analyzed with ImageJ.

### 4.14 Co‐Immunoprecipitation and Mass Spectrometry Assays

For co‐immunoprecipitation (co‐IP), cells overexpressing Flag‐tagged GSTA4 were lysed, and whole‐cell extracts were incubated overnight at 4°C with protein A/G magnetic beads (MCE, HY‐K0202). After extensive washing, bound proteins were eluted and analyzed by immunoblotting to detect interacting proteins. For mass spectrometry, immunoprecipitated samples were subjected to acetone precipitation. Precipitated proteins were digested with trypsin, and the resulting peptides were resuspended, centrifuged, and analyzed by LC‐MS/MS on a Q Exactive HF‐X mass spectrometer (Thermo Scientific). Protein identification and interaction profiling were performed using MaxQuant against the UniProt human database.

### 4.15 Pull‐Down Assay

Recombinant GST‐ and Flag‐tagged proteins were expressed and purified from HEK293T cells. For pull‐down assays, GST‐tagged proteins immobilized on glutathione magnetic agarose beads (MCE, HY‐K0234) were incubated with Flag‐tagged proteins overnight at 4°C. After extensive washing, bound complexes were eluted and analyzed by SDS‐PAGE followed by Western blot.

### 4.16 Molecular Docking, Virtual Screening, and Molecular Dynamics Simulations

Molecular docking was performed using AutoDock Vina to predict binding modes and affinities between ligands and the target protein. For virtual screening, an FDA‐approved drug library was screened in silico, and compounds with well‐defined established targets were excluded from subsequent analysis. Molecular dynamics simulations were conducted using GROMACS to assess the stability and conformational dynamics of the protein‐ligand complexes over 100 ns trajectories.

### 4.17 Bioinformatics Analysis

RNA sequencing was performed on C4‐2 parental and C4‐2‐ENZR cells (Xurangene Biotechnology). DEGs analysis was conducted using DESeq2, and functional enrichment was assessed via ssGSEA. Publicly available RNA‐seq datasets related to ENZR samples were obtained from the GEO database. DEGs were identified and subjected to KEGG enrichment analysis. Further ssGSEA was carried out using a ferroptosis‐related gene set. For scRNA‐seq analysis, data from the GSE206962 dataset (ENZR PCa) were processed. Cell types were annotated based on canonical marker genes using Seurat. Subsequent analyses included differential expression and subpopulation characterization.

### 4.18 Surface Plasmon Resonance (SPR)

SPR was performed to characterize the binding interaction between human GSTA4 protein and metformin hydrochloride using a Biacore system. The CM5 sensor chip (Cytiva) was functionalized with human GSTA4 via standard amine coupling chemistry. Briefly, the chip surface was activated with a mixture of 1‐ethyl‐3‐(3‐dimethylaminopropyl)carbodiimide (EDC) and N‐hydroxysuccinimide (NHS), followed by injection of 50 µg/mL protein solution in acetate buffer (pH 4.5). Remaining reactive groups were blocked with ethanolamine. A reference flow cell, prepared under identical conditions but without protein immobilization, was used for background subtraction. Binding analyses were conducted by injecting metformin hydrochloride at a range of concentrations over the protein surface at a flow rate of 30 µL/min for 150 s, followed by a 5‐minute dissociation phase. The sensor surface was regenerated with 10 mM glycine‐HCl (pH 2.0) after each cycle to ensure complete removal of bound analyte. All sensorgrams were processed and evaluated using Biacore Insight evaluation software. Binding kinetics including the association rate constant (Ka), dissociation rate constant (Kd), and equilibrium dissociation constant (K_D_) were determined by globally fitting the data to a 1:1 Langmuir binding model.

### 4.19 Statistics

Data are presented as mean ± SD. Statistical analyses were performed using GraphPad Prism 10 and SPSS 26.0. Group comparisons were analyzed by two‐tailed unpaired Student's t‐test (two groups) or one‐way ANOVA (multiple groups). Survival curves were compared using the log‐rank test. Univariate and multivariate Cox proportional hazards models were employed to assess independent prognostic factors. *P* < 0.05 was considered statistically significant.

## Author Contributions

Q.Y., S.P., and Y.L. (**Yong Luo**) conceived the study, designed and performed the majority of the experiments, analyzed the data, and drafted the manuscript. W.Z., Z.L., and Y.L. (**Yiming Lai**) were responsible for clinical sample collection, data curation, and providing essential experimental support and reagents. Y.O., D.L., S.H., T.L., J.H., B.C., J.F., T.Z., H.J., and Y.L. (**Yin Lu**) provided expert consultation, and essential feedback on the manuscript. Q.Y., S.P., K.X., H.H., Y.L. (**Yiming Lai**), and Z.L. provided overall study supervision, managed project administration and funding acquisition, and critically reviewed and edited the final manuscript. All authors reviewed and approved the final version of the manuscript.

## Use of Generative AI and AI‐Assisted Technologies in the Writing Process

No generative artificial intelligence (AI) tools were used in the preparation of the manuscript text, figures, or graphical abstract.

## Conflicts of Interest

The authors declare no conflicts of interest.

## Supporting information




**Supporting File**: advs77908‐sup‐0001‐SuppMat.docx.

## Data Availability

The data that support the findings of this study are available from the corresponding author upon reasonable request.
