# Supplementary material for: Dual Compartmentalization of GSTA4 Suppresses Ferroptosis to Drive Antiandrogen Resistance in Prostate Cancer
Source: Adv Sci (Weinh). 2026 Sep 27:e77908. Online ahead of print. doi: 10.1002/advs.77908 (PMC13616357; doi:10.1002/advs.77908)
Supplement: Supplementary file 1 [file ADVS-9999-e77908-s001.docx]

**Figure S1 Negative regulators of ferroptosis are key determinants of resistance to ENZ in CRPC, related to Figure 1.**

(A-G) Volcano plots displaying DEGs from transcriptomic analyses of ENZR cell lines, including in-house data and six public GEO datasets (GSE70380, GSE78201, GSE104935, GSE151083, GSE169305, and GSE202885). (H-M) Functional enrichment analysis of ENZR cell transcriptomes from GSE70380, GSE78201, GSE104935, GSE151083, GSE169305, and GSE202885. (N, O) Basal levels of 4-hydroxynonenal (4-HNE) (N) and intracellular glutathione (GSH) (O) were measured in ENZ-sensitive and ENZ-resistant cells and normalized to cell number. (P) Body weight changes of mice during the xenograft experiments corresponding to Figure 1I-L. (Q, R) *In vitro* assessment demonstrated that co-treatment with ENZ and ferroptosis inhibitors confers resistance to ENZ.

**Figure S2 GSTA4 is upregulated in ENZR PCa and associated with reduced lipid peroxidation, related to Figure 2.**

(A) Radar plot shows the fold change of GSTA4 expression in ENZR versus sensitive groups across six GEO datasets (GSE70380, GSE78201, GSE104935, GSE151083, GSE169305, and GSE202885). (B) UMAP plot of cell types from human ENZR PCa samples (GSE206962). (C) UMAP plots illustrated GSTA4 expression distribution across cell types in ENZ-sensitive (left) and ENZ-resistant (right) samples. (D, E) Bar plot (D) and expression distribution (E) of GSTA4 in tumor cells from ENZ-sensitive versus ENZ-resistant groups in human PCa scRNA-seq data. (F) Heatmap of GSTA4 expression from RNA sequencing in our ENZR cells. (G) qPCR validation of GSTA4 upregulation in ENZR cells. (H) ENZR CRPC mouse model established using *Pten/Trp53* double-knockout mice, wherein resistant tumors exhibited significantly larger volumes. (I) IHC staining of 4-HNE in ENZ-sensitive and ENZ-resistant spontaneous mice models. Scale bars: 200 μm (main), 50 μm (inset). (J) The orthotopic ENZR CRPC model established with *Pten/Trp53*-deficient prostate organoids, exhibiting increased tumor growth. (K) IHC staining of 4-HNE in ENZ-resistant and ENZ-sensitive orthotopic organoid models. Scale bars: 200 μm (main), 50 μm (inset). (L-N) Bar graphs showed quantitative expression of 4-HNE (L) and GSTA4 (M), and correlation analysis indicated a negative relationship between GSTA4 and 4-HNE levels (N) in spontaneous ENZR CRPC model. Scale bars: 200 μm (main), 50 μm (inset). (O-Q) Bar graphs show quantitative expression of 4-HNE (O) and GSTA4 (P) and correlation analysis demonstrated a reciprocal relationship between GSTA4 and 4-HNE levels (Q) in orthotopic ENZR CRPC model.

**Figure S3 GSTA4-mediated evasion of ferroptosis underlies ENZ resistance in PCa, related to Figure 3.**

(A, C) Cell viability assays in C4-2 and VCaP cells with GSTA4-KD treated with RSL3 (A) or erastin (C), showing rescue from cell death specifically by ferroptosis inhibitors. (B, D) Dose-response viability curves of GSTA4-KD C4-2 and VCaP cells treated with different doses of RSL3 (B) or erastin (D), in the presence or absence of Lip-1. (E) Western blot analysis validating GSTA4-KD efficiency in C4-2 and VCaP cells. (F) Western blot analysis of GSTA4 expression in wild-type cells treated with increasing concentrations of ENZ. (G) Fluorescence images showing increased lipid peroxidation in ENZR cells with GSTA4-KD upon ENZ treatment. Scale bar, 20 μm. (H) GSTA4-OE significantly decreased lipid peroxidation, indicated by reduced green fluorescence and enhanced red fluorescence. Scale bar, 20 μm. (I, J) Body weight changes of mice bearing ENZR xenografts corresponding to Figure 3L and Figure 3N. (K) Representative IHC staining images of 4-HNE in ENZR cell-derived xenografts. Scale bar, 50 μm. (L) Representative IHC staining images of 4-HNE in GSTA4-OE xenografts following ENZ treatment. Scale bar, 50 μm.

**Figure S4 GSTA4 mediates ferroptosis defense independently of the canonical pathways, related to Figure 4.**

(A) Cell viability assays of C4-2 and VCaP cells with GSTA4-KD treated with Lip-1 upon erastin treatment, with concurrent knockdown of GPX4, FSP1, or DHODH. (B, C) Western blot analysis of GSTA4 expression in C4-2 and VCaP cells upon FSP1-KD (B) or DHODH-KD (C).

**Figure S5 GSTA4 interacts with PGAM5 to coordinate mitochondrial redox balance, related to Figure 5.**

(A) Functional enrichment analysis of scRNA-seq data from GSTA4-high vs. GSTA4-low cells, showing terms associated with mitochondrial function and redox homeostasis. (B) Mass spectrometry dataset of GSTA4 immunoprecipitates upon erastin treatment. (C) Mass spectrometry analysis identifying PGAM5 as an interaction partner of GSTA4. (D) Molecular docking model of the interaction between GSTA4 (yellow) and PGAM5 (purple).

**Figure S6 GSTA4 regulates Drp1 mitochondrial translocation under erastin treatment, related to Figure 6.**

(A, B) Confocal microscopy images showing GSTA4 WT and Δ1 inhibit erastin-induced mitochondrial translocation of Drp1, while the GSTA4 Δ2 mutant does not, in C4-2 (A) and VCaP (B) cells. Scale bar, 10 μm.

**Figure S7 Targeting GSTA4-mediated antioxidant defense by metformin reverses ENZ resistance, related to Figure 7.**

(A, B) Cell viability assays in GSTA4-KD ENZR cells (A) and GSTA4-OE VCaP cells (B) treated with atorvastatin (Ato), celecoxib (CBX), or metformin (Met). (C-F) Molecular dynamics simulations of the metformin-GSTA4 complex, showing RMSD (C), radius of gyration (D), RMSF (E), and SASA (F) profiles. (G) Western blot analysis of GSTA4 expression in ENZ-sensitive (Sen) and ENZ-resistant (Res) cells treated with metformin. (H) Concentration-dependent effect of metformin on GSTA4 protein levels in C4-2 and VCaP cells, assessed by western blot. (I) Representative western blot showing p-AMPK, AMPK, GSTA4, and α-tubulin in C4-2 and VCaP cells treated with metformin in the presence or absence of dorsomorphin. (J, K) Colony formation assays in (J) ENZR and GSTA4-OE VCaP (K) cells under under metformin and ENZ treatment. (L) Body weight changes of mice during the subcutaneous xenograft study corresponding to Figure 7G-J. (M) IHC staining images of 4-HNE in xenograft tumors from different treatment groups and corresponding quantitative scoring. Scale bar, 50 μm.

**Table S1**. Comparison of Baseline Clinicopathological Characteristics Between HSPC and CRPC Patients

| **Characteristic** | **Overall (N=158)** | **HSPC (N=140)** | **CRPC (N=18)** | ***P* value** |
| --- | --- | --- | --- | --- |
| **Age** |  |  |  | 0.213 |
| <70 years | 77 (48.7%) | 71 (50.7%) | 6 (33.3%) |  |
| ≥70 years | 81 (51.3%) | 69 (49.3%) | 12 (66.7%) |  |
| **Tumor grade** |  |  |  | **0.006** |
| Grade 1 | 11 (7.0%) | 10 (7.1%) | 1 (5.6%) |  |
| Grade 2 | 53 (33.5%) | 52 (37.1%) | 1 (5.6%) |  |
| Grade 3 | 34 (21.5%) | 32 (22.9%) | 2 (11.1%) |  |
| Grade 4 | 27 (17.1%) | 21 (15.0%) | 6 (33.3%) |  |
| Grade 5 | 33 (20.9%) | 25 (17.9%) | 8 (44.4%) |  |
| **T stage** |  |  |  | **<0.001** |
| T1–T2 | 80 (50.6%) | 78 (55.7%) | 2 (11.1%) |  |
| T3–T4 | 78 (49.4%) | 62 (44.3%) | 16 (88.9%) |  |
| **N stage** |  |  |  | **0.028** |
| N0 | 129 (81.6%) | 117 (83.6%) | 12 (66.7%) |  |
| N1 | 22 (13.9%) | 16 (11.4%) | 6 (33.3%) |  |
| Nx | 7 (4.4%) | 7 (5.0%) | 0 (0.0%) |  |
| **M stage** |  |  |  | **0.019** |
| M0 | 141 (89.2%) | 127 (90.7%) | 14 (77.8%) |  |
| M1 | 10 (6.3%) | 6 (4.3%) | 4 (22.2%) |  |
| Mx | 7 (4.4%) | 7 (5.0%) | 0 (0.0%) |  |
| **tPSA** |  |  |  | **0.013** |
| <20 ng/mL | 109 (69.0%) | 102 (72.9%) | 7 (38.9%) |  |
| ≥20 ng/mL | 44 (27.8%) | 34 (24.3%) | 10 (55.6%) |  |
| Unknown | 5 (3.2%) | 4 (2.9%) | 1 (5.6%) |  |
| **fPSA** |  |  |  | **0.003** |
| <10 ng/mL | 115 (72.8%) | 101 (72.1%) | 14 (77.8%) |  |
| ≥10 ng/mL | 6 (3.8%) | 3 (2.1%) | 3 (16.7%) |  |
| Unknown | 37 (23.4%) | 36 (25.7%) | 1 (5.6%) |  |

|  | Gene names | Sequences |
| --- | --- | --- |
| shRNA | GSTA4-1 | ACAACAUCUUUAGGCCAUATT |
|  | GSTA4-2 | ACAAUCUCUUUGGCAAGAATT |
|  | GPX4 | GTGGATGAAGATCCAACCCAA |
|  | FSP1 | CAATGAGTATCGAGAGTACAT |
|  | DHODH | TCCGGGATTTATCAACTCAAA |
| RT-PCR primer | GSTA4 | Forward: AAGGAGAGAACCCTGATTGACA |
|  |  | Reverse: GTCCGTGACCCCTTAAAATCTTT |

**Table S2**. The shRNA and RT-PCR primer sequences of this manuscript
